# Genetic evidence supports three previously described species of greater glider, *Petauroides volans*, *P. minor*, and *P. armillatus*

**DOI:** 10.1038/s41598-020-76364-z

**Published:** 2020-11-06

**Authors:** Denise C. McGregor, Amanda Padovan, Arthur Georges, Andrew Krockenberger, Hwan-Jin Yoon, Kara N. Youngentob

**Affiliations:** 1 College of Science and Engineering, James Cook University, Cairns, QLD 4878 Australia; 2 CSIRO Black Mountain Science and Innovation Park, Canberra, ACT 2601 Australia; 3 Institute for Applied Ecology, University of Canberra, Canberra, ACT 2601 Australia; 4 Division of Research and Innovation, James Cook University, Cairns, QLD 4878 Australia; 5 Statistical Consulting Unit, Australian National University, Canberra, ACT 2601 Australia; 6 Research School of Biology, Australian National University, Robertson Building, 46 Sullivan’s Creek Road, Canberra, ACT 2601 Australia

**Keywords:** Conservation biology, Genomics

## Abstract

The identification and classification of species are essential for effective conservation management. This year, Australia experienced a bushfire season of unprecedented severity, resulting in widespread habitat loss and mortality. As a result, there has been an increased focus on understanding genetic diversity and structure across the range of individual species to protect resilience in the face of climate change. The greater glider (*Petauroides volans*) is a large, gliding eucalypt folivore. This nocturnal arboreal marsupial has a wide distribution across eastern Australia and is considered the sole extant member of the genus *Petauroides*. Differences in morphology have led to suggestions that the one accepted species is actually three. This would have substantial impacts on conservation management, particularly given a recent history of declining populations, coupled with extensive wildfires. Until now, genetic evidence to support multiple species has been lacking. For the first time, we used DArT sequencing on greater glider tissue samples from multiple regions and found evidence of three operational taxonomic units (OTUs) representing northern, central and southern groups. The three OTUs were also supported by our morphological data. These findings have important implications for greater glider management and highlight the role of genetics in helping to assess conservation status.

## Introduction

Effective conservation management relies on accurate taxonomic classification and a robust understanding of the genetic structure of populations. A lack of knowledge about the genetic structure of species across their range can result in an inability to properly manage and protect species from extinction^1^. This is especially true in the wake of a natural disaster, when wildlife management decisions need to be made quickly and under challenging circumstances.

The catastrophic 2019–2020 bushfire season in Australia burnt over 97,000 km^2^ and directly or indirectly killed millions of native animals^2^. The impacts of fire on genetic diversity are nontrivial^3^, and the extent of the recent fires means that substantial portions of many species’ ranges were impacted^2^. As a result, the conservation status of a number of species are being revisited and the importance of protecting genetic diversity within species is becoming more widely recognised for its central role in conserving species’ resilience to anthropogenic climate change^4–6^.

**Figure 1:**
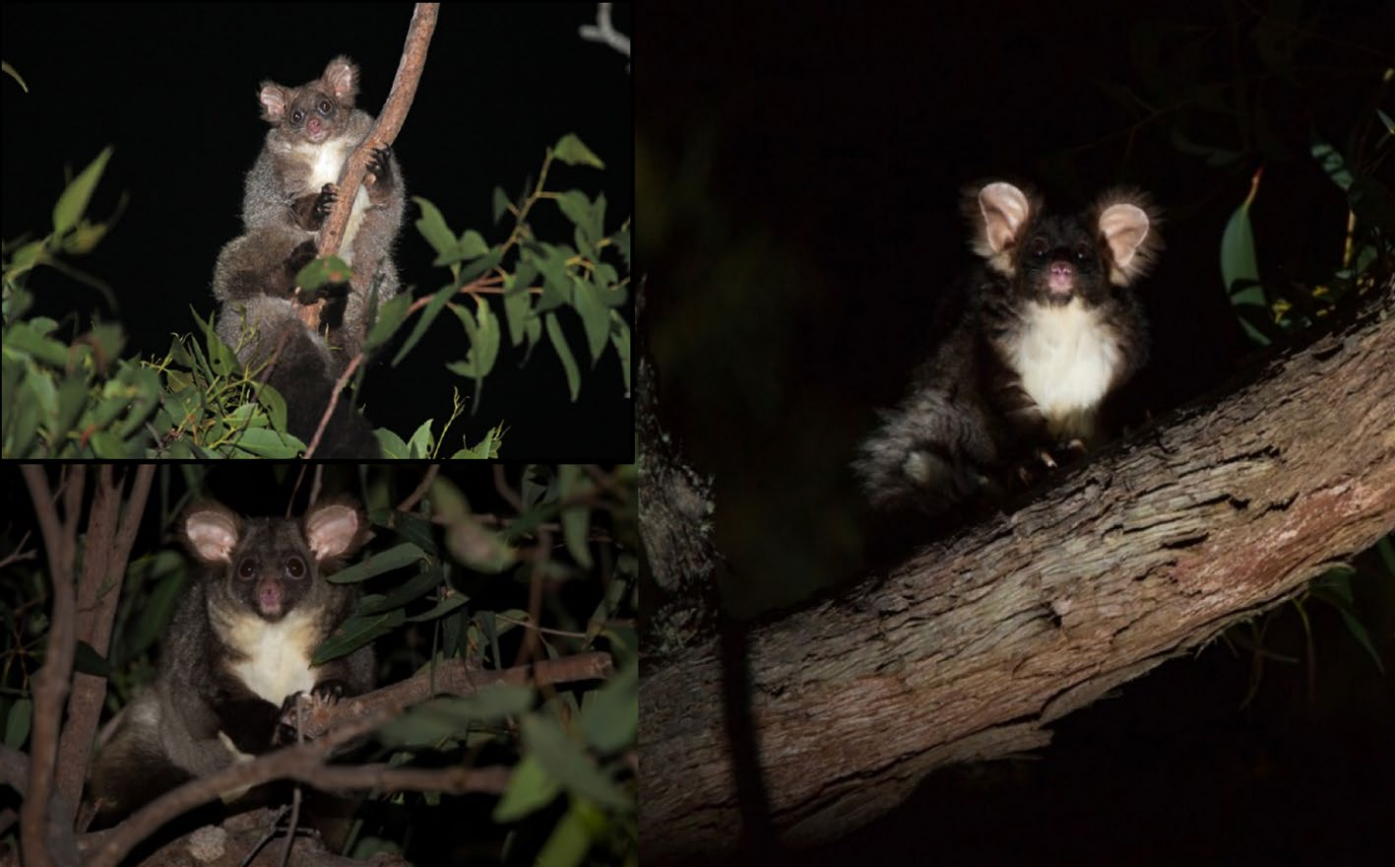
Greater gliders from the northern (top left), central (bottom left) and southern (right) groups identified through DArTseq showing morphological differences that are typical of our dataset. Greater gliders of the type shown on the right have several pelage colour morphs including white and light grey. Photos by Denise McGregor (top left) and Jasmine Vink (bottom left and right).

The greater glider (*Petauroides volans*, (Kerr^7^) is a large, nocturnal gliding marsupial endemic to Australia (Fig. 1). Strictly arboreal, they are vulnerable to habitat loss and disturbance due to their specialised diet of eucalypt leaves and obligate dependence on mature trees with large hollows for shelter^8^. Greater gliders have a widespread distribution primarily associated with eucalypt forests along the Great Dividing Range from northern Queensland to southern Victoria^9^.

Data from long-term monitoring sites have revealed alarming declines and localized population extinctions of greater gliders over the past twenty years^9–12^. As a result, this once common and abundant species is now listed as vulnerable under the National Environmental Protection and Biodiversity Conservation Act^13^ and vulnerable globally under the IUCN’s Red List of threatened species^14^. Owing to the recent widespread fires and the history of unresolved population declines, the conservation status of the currently recognised single species, *P. volans*, is being reconsidered. The division of the greater glider into multiple species would have substantial conservation implications. In particular, population declines and recent fire impacts, which are mostly associated with populations south of Queensland, may extend across a much greater proportion of the distribution of *P. volans* than previously thought, and have a more substantial combined effect.

*Petauroides volans* is regarded as the sole member of the genus *Petauroides* and the only gliding member of the family Pseudocheiridae^13,14^. There are currently two subspecies of *P. volans* recognised by the Australian Government for the purpose of conservation legislation, *P. v. minor* and *P. v. volans*^13^. They have been described on the basis of morphological differences in body size, pelage colouration, and geographic distribution^15,16^. *Petauroides volans volans* is found south of the Tropic of Capricorn, weighs between 1000–1700 g and occurs in several colour morphs ranging from completely black, to black with a white belly and chest, to lighter grey dorsal pelage or white all over^15,17^. *Petauroides volans minor* has a relatively small body mass of 650–1100 g, occurs from the Tropic of Capricorn to just north of Cairns, and also has light ventral pelage, but the dorsal hair has dark brown tips and lighter subterminal bands^16,18^.

Recently, Jackson^19^ and Jackson and Groves^20^ recognised *P. volans* as comprising three distinct species, geographically delineated across the range of *Petauroides*. This raises the subspecies *P. volans minor*, to species status (*P. minor*) and resurrects the name *P. armillatus*, to apply to populations in mid-Queensland from the Eungella Range to just north of Townsville^19^. *Petauroides armillatus* was once considered a subspecies (*P. volans armillatus*) by Thomas in 1923^21^ but subsequently synonymized within minor^22^. The multi-species taxonomy of Jackson^19^ and Jackson and Groves^20^ retains *Petauroides volans* as the most southerly distributed species from Bundaberg in Queensland (Qld) to Victoria (Vic), and includes two subspecies (*P. volans incus* and *P. volans volans*).

The claim that the three taxa *P. volans*, *P. minor* and *P. armillatus* are morphologically and genetically distinct at the level of species has been controversial because it lacks supporting published evidence and was referenced by Jackson and Groves^20^ from a personal communication with the late Dr Kenneth Aplin and a brief conference abstract by Arbogast et al.^23^. Neither of these sources have published data to support the distinctiveness of the proposed *P. minor*, *P. armillatus*, and *P. volans*. For the first time, we test Jackson and Groves’s^20^ three species designation using molecular sequence data, DArTseq^24^, and report the genetic population structure of greater gliders at multiple points across their natural range.

## Results

### Summary of morphological data

**Figure 2:**
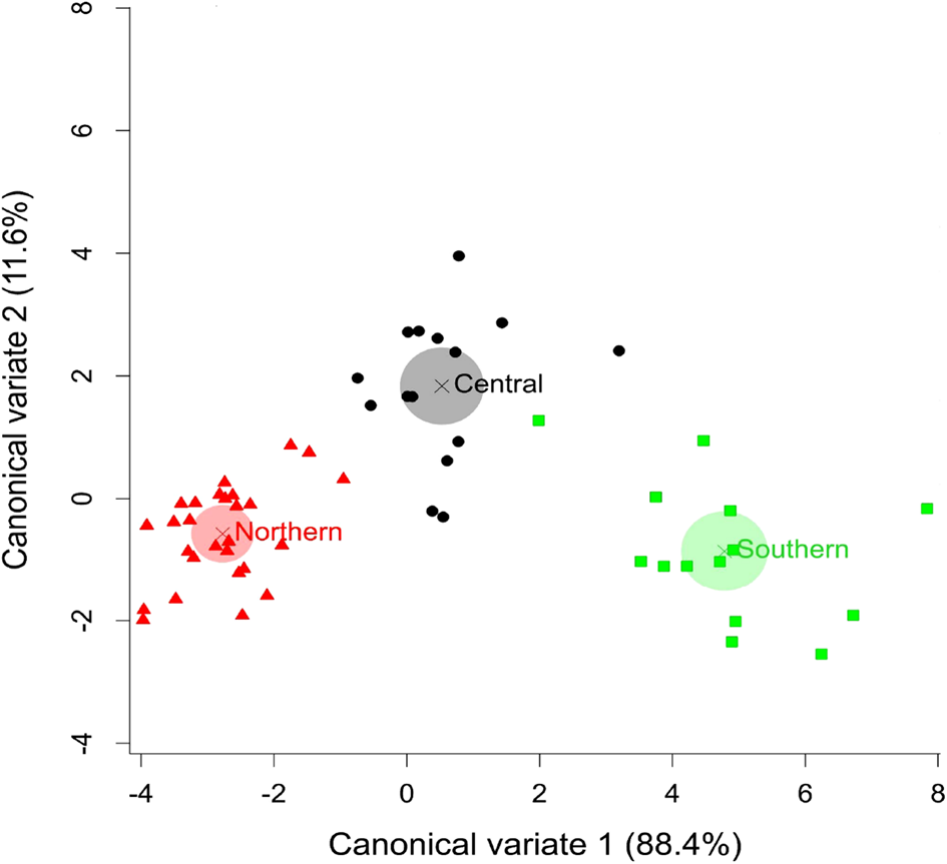
Canonical variate analyses (CVA) of body measurements of 56 greater gliders from three regions (Northern, Central, and Southern). The shaded circles are the group mean.

We collected eight morphological measurements from 63 greater gliders sampled from five locations (Supplementary materials Table S1 and S2 available online). Morphological measurements were not available for the 12 museum samples (Supplementary Table S3). Only adult animals were used in the morphological analyses, resulting in 56 individuals. Data on sex based differences from other studies are equivocal^18,25^. Using linear models for unbalanced data with backward elimination method, we found that measurements for males and females in our dataset were not significantly different (*p* > 0.05) in seven out of eight attributes, with the exception that females had longer tails (F_1,52_ = 7.14, *p* = 0.01). We combined male and female data within locations for the remaining analyses. A principal components analysis (PCA) of morphological measurements broadly clustered into three groups with two samples showing some overlap (PC1 = 70%, PC2 = 8.3%, Supplementary Fig. S1 and Table S4). Body mass was the strongest contributor to differences among groups (Supplementary Table S4 and Fig. S1). A canonical variates analysis (CVA) of the measured traits from the three groups representing samples collected from the northern sites (Taravale and Blackbraes), central site (Redcliffe Vale), and southern sites (Bendoc and Wombat) demonstrated a high degree of separation (Fig. 2, CV1 = 88.4% and CV2 = 11.6%).

We further investigated differences between greater glider morphology across locations using Tukey’s post-hoc multiple comparison test. We found no significant difference in greater glider body measurements between the two southern sites (Wombat and Bendoc) for any of the eight measured attributes (Supplementary Table S5). Similarly, greater gliders from the two northern sites (Taravale and Blackbraes) differed only in that Taravale gliders had significantly longer tails (t_54_ = 4.99, *p* < 0.0001, Supplementary Table S5). We then collapsed the two southern sites into a single region called Southern and the two northern sites into a single region called Northern, and explored morphological differences in greater gliders among the three regions (Northern, Central, and Southern; summary statistics provided in Table 1). The Northern and Southern greater gliders differed significantly in every measured morphological attribute (Table 2). Northern and Central greater gliders differed significantly in every measured attribute except body length (t_54_ = − 0.23, *p* = 0.971), and Central and Southern greater gliders differed significantly in every attribute except ear length (t_54_ = − 5.92, *p* = 0.502) and ear width (t_54_ = 0.97, *p* = 0.595; Table 2). Northern greater gliders were the smallest of the three groups in almost every measured attribute and Southern greater gliders were the largest, with the Central intermediate, but still significantly different in most measured attributes from the Northern and Southern groups. Descriptions of pelage colouration, which was distinctly different between regions, can be found in the supplementary materials (Supplementary Table S2 and Fig. S2).

**Table 1:** Means and (standard deviations) of the measured morphological traits from sampled greater gliders across three regions. Mass unit is in kilograms, body length and tail length are measured in centimetres and all other measured traits are in millimetres.

Region	N	Mass	Head length	Head width	Body length	Tail length	Knee to heal	Ear length	Ear width
Northern	27	0.68 (0.06)	57.21 (2.99)	34.19 (1.61)	27.89 (2.47)	40.78 (3.23)	104.19 (4.91)	35.9 (2.06)	26.57 (3.49)
Central	15	0.87 (0.09)	64.17 (3.21)	37.26 (1.51)	28.27 (3.31)	45.02 (2.13)	112.59 (5.02)	40.37 (4.02)	29.88 (2.87)
Southern	14	1.36 (0.16)	67.05 (2.34)	39.14 (2.79)	36.11 (2.95)	51.75 (2.82)	121.04 (3.82)	41.62 (3.28)	31 (2.46)

**Table 2:** Tukey’s Post-hoc test between regions for each measured morphological trait.

Comparison	Northern-Central			Central-Southern			Northern–Southern		
	Diff. in mean (SE)	*t* value	*p* value	Diff. in mean (SE)	*t* value	*p* value	Diff. in mean (SE)	*t* value	*p* value
Body mass	− 0.19 (0.03)	− 5.66	****	0.50 (0.04)	13.13	****	0.68 (0.03)	20.35	****
Head length	− 6.95 (0.94)	− 7.43	***	2.88 (1.08)	2.67	*	9.83 (0.96)	10.27	***
Head width	− 3.08 (0.63)	− 4.91	****	1.88 (0.72)	2.59	*	4.95 (0.64)	7.72	****
Body length	− 0.21 (0.91)	− 0.23	0.97	7.84 (1.05)	7.45	****	8.05 (0.93)	8.63	****
Tail length	5.65 (0.93)	− 6.10	****	6.73 (1.07)	6.31	****	12.38 (0.95)	13.09	****
Knee to heel	− 10.77 (1.51)	− 7.11	****	8.45 (1.75)	4.84	****	19.21 (1.55)	12.41	****
Ear length	− 5.71 (0.97)	− 5.92	****	1.25 (1.11)	1.12	0.50	6.96 (0.99)	7.06	****
Ear width	− 4.31 (1.00)	− 4.32	***	1.12 (1.15)	0.97	0.60	5.44 (1.02)	5.32	****

****p < 0.0001, ***p < 0.001, **p < 0.01, *p < 0.05.

### Summary of geographic population structuring and metadata

Genetic population structure analysis revealed three diagnosable aggregations that we refer to as operational taxonomic units (OTUs). The raw data consisted of 78 individuals scored for SNP polymorphisms at 45,245 loci. Filtering, as outlined in Methods, resulted in only high-quality SNP loci being retained with high coverage across individuals and loci, ensuring completeness of the final dataset and minimising the amount of missing data. This yielded SNP calls for 11,317 loci for 75 individuals. Following this filtering, these data are considered to be highly reliable.

**Figure 3:**
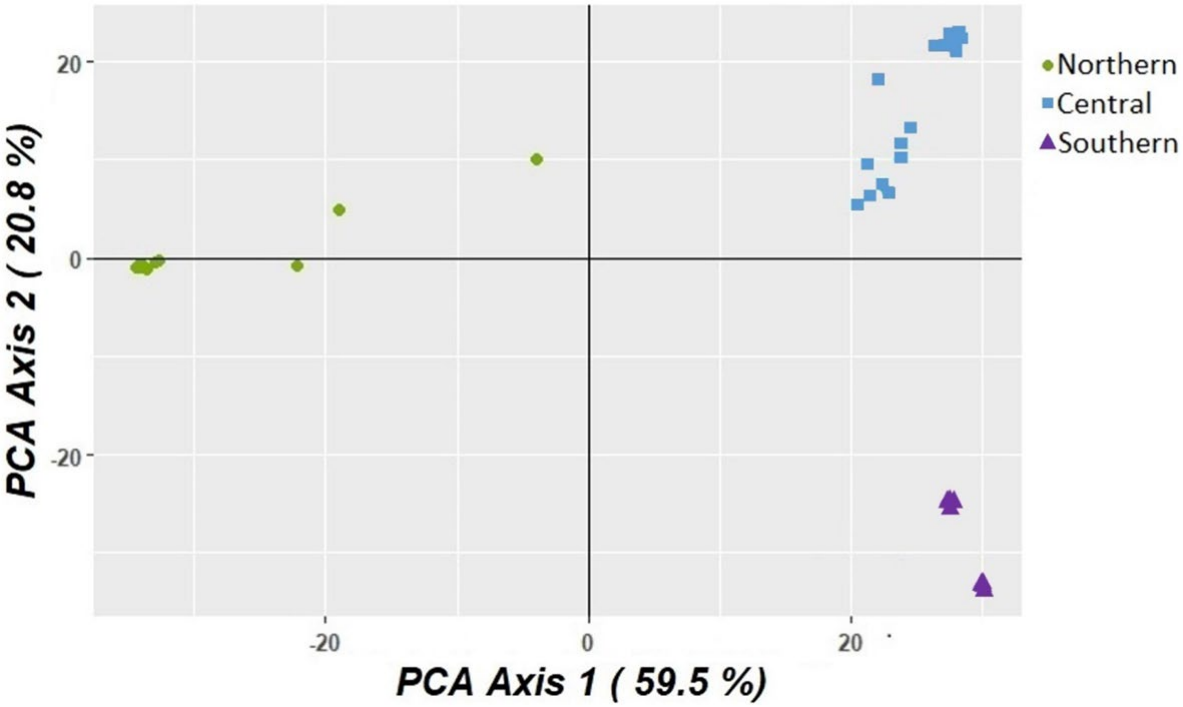
Principal component analysis (PCA) of genetic data coloured by collapsed sample regions. PC1 explains 59.5% of the variation in the dataset and PC2 explains 20.8% of the variation. The potential hybrids are the samples from the Northern group that are plotted between that group and the Central group.

Principal components analysis of this dataset revealed three distinct genetic groups corresponding to geographic locations of Taravale/Blackbraes (Northern), Bendoc and Wombat (Southern) and Redcliffe Vale/Museum (Central) (Fig. 3). Two individuals from Taravale (Northern) lay intermediate in position between the Northern cluster and the Central cluster, and are likely instances of contemporary hybridization, a proposition that we test in a separate analysis.

Excluding the putative hybridizations, fixed differences analysis^26,27^ identified four putative operational taxonomic units (OTUs) diagnosable on the basis of one or more fixed allelic differences—the Northern (Taravale and Blackbraes combined), and the two Southern, Bendoc and Wombat. Wombat (n = 6) and Bendoc (n = 9) were distinguished by only 2% (233) fixed differences in comparison with the other populations that differed between 11 and 30% (1373–4767). Test of significance demonstrated that the difference between Wombat and Bendoc was not significant (i.e. did not exceed the false positive rate for fixed differences, given the respective sample sizes—see Georges et al.^26^: R scripts are provided in the online data repository). We therefore combined Wombat and Bendoc to yield a final three putative OTUs, representing North, Central, and Southern populations (Fig. 2). Percent fixed differences between OTUs are presented in Table 3 and counts of private alleles between the final OTUs taken pairwise are presented in Table 4.

**Table 3:** Percent fixed differences between populations taken pairwise (lower matrix and diagonal, loci = 11,317) and average number of individuals scored (upper matrix) to estimate the fixed differences. Note that Taravale and Blackbraes have no fixed allelic differences and so are amalgamated into a single OTU (Northern); the 2% fixed difference between Bendoc and Wombat were not statistically significant and so were amalgamated into a single OTU (Southern).

Sites	Bendoc	Blackbraes	Redcliffe	Taravale	Wombat
Bendoc	0	24	26.7	25	14.9
Blackbraes	41	0	32.7	31	20.9
Redcliffe	17	26	0	33.7	23.7
Taravale	39	0	23	0	21.9
Wombat	2	33	10	31	0

**Table 4:** Private alleles between final OTUs, taken pairwise, for OTUs vertically listed (lower matrix)) and horizontally listed (upper matrix). This is based on 11,317 loci, that is, post filtering. We did not filter on minor allele frequency. The count of private alleles includes the count of fixed differences.

	Northern	Central	Southern
Northern	0	4307	5149
Central	5717	0	4035
Southern	6527	4003	0

Southern greater gliders had the highest expected heterozygosity (0.11), a measure of genetic diversity, substantially higher than Northern gliders (0.04, *p* < 0.0001) and marginally higher than Central gliders (0.10, *p* < 0.02). Northern gliders had the lowest heterozygosity (0.04), significantly lower also than Central gliders (0.10, *p* < 0.0001).

### Hybrids exist between the two Queensland genetic groups

The two individuals collected at Taravale (Northern group) had intermediate positions in the PCA falling between the Northern group and the Central group. Comparing these individuals with likelihood bins associated with parental genotypes, F1 hybrids, backcrosses of the F1 to the parentals, and F2 hybrids using New Hybrids^28^ showed that one individual had a genotype consistent with an F1 hybrid between the Northern group and the Central group (T1) and the other a genotype consistent with a backcross of an F1 hybrid to the Northern group (T5) (Table 5). We suspected based on the proximity of capture, ages, and evidence of an F1 and backcross genotypes, that individuals T1 and T5 might be parent-offspring. Analysis of pedigree incompatibility estimates for this pair and pairs taken at random from our pool of individuals, indicated that a parent–offspring relationship between T1 and T5 was highly likely. The parental assignments arose naturally from the analysis, and were not defined a priori. The analysis identified one F1 (T1) and one F1 × P0 (T5).

**Table 5:** Results of the New Hybrids analysis. IDs, the identifiers used for each individual glider; P0, parental population Northern; F1, F1 hybrid; F2, F2 hybrid; F1 × P0, backcross of an F1 with the Northern parental population; F1 × P1, backcross of an F1 with the Central parental population. Count is the number of individuals tallied for each class. The body of the table contains the posterior probabilities of group membership, calculated from the likelihood distributions generated by simulation by New Hybrids^28^.

IDs	Pop	Count	P0	P1	F1	F2	F1 × P0	F1 × P1
T5	Northern	1	0	0	0	0	1	0
T1	Northern	1	0	0	1	0	0	0
T2, B9, T3, B2, B4, T17, B12, T7, B14, T18, B13, B3, T15, B5, T9, B1, T6, T11, B7, T14, T10, T4, Bjuv, T13, T12, T16, B15, B10, T8, B6, B8, MS2	Northern	32	1	0	0	0	0	0
ER1. E9. ER17, ER2, ER10, ER10A, ER3, ER11, ER4, ER12, ER5, ER13, ER6, ER14, ER7, ER15, ER8, ER16, MS3, MS7, MS1, MS10, MS11, MS4, MS5, MS6	Central	26	0	1	0	0	0	0

## Discussion

Greater gliders are currently classified as a single species, *P. volans*^13,14^. We used DArTseq on samples from multiple locations across the range of *P. volans*, which revealed three operational taxonomic units (OTUs) representing Northern, Central and Southern groups. These broadly correspond with the species designations summarised by Jackson and Groves^20^ and provide the first genetic support for multiple greater glider species. The morphological measurement from the sampled greater gliders from the five study areas also align with these three OTUs. The Northern and Southern populations differed significantly in all morphological measurements, and this is in keeping with previously reported differences in the ear, body size, and pelage colouration of the currently recognised subspecies (*P. volans minor* and *P. volans volans*)^16^. There was some overlap between animals from the Central population in Redcliffe Vale and the other two groups in morphology, but the Central population still differed significantly in a number of measurements from the Northern and the Southern animals. We also found evidence of hybridisation between the Northern and Central groups and one of those hybrids had morphological measurements that were intermediate between those groups as well. As discussed by de Queiroz^29^, delimiting species is complicated by the numerous species concepts. The Genetic Species Concept recognises that species are genetically isolated but not reproductively isolated, allowing for hybridisation, which is likely to occur during the speciation process^30^.

*Petauroides volans* has a long and complicated taxonomic history, which has been extensively reviewed by Maloney^31^ and Maloney and Harris^32^. The greater glider was first identified to western science by Kerr^7^, with further variability in morphology among collected individuals described by Collett^18^ who suggested a “variety” called *Petauroides volans minor* based on differences in skull size, body length and pelage colour. The greater glider was more formally described as one species by Oldfield Thomas^21^ with four subspecies (*Petauroides volans typicus*, *P. v. minor*, *P. v. incanus* and *P. v. armillatus*)^31^. *Petauroides v. armillatus* was then synonymised with *P. v. minor* in 1934 by Iredale and Troughton who also changed the genus to *Schoinobates* resulting in three renamed subspecies *S. v. minor*, *S. v. incanus*, and *S. v. volans* (previously *typicus*)^19,20^. *Petauroides* was restored by McKay in 1982 who also synonymised *P. volans incanus* with *P. v. volans* in 1988^20,22^. Based on personal communications with the late Dr Kennith Aplin and unpublished data in a conference presentation by Arbogast et al.^23^, Jackson and Groves^19,20^ recognised three species within *Petauroides* and two subspecies. Until now, there has been no published genetic evidence to support either multiple or single species designations, and *P. volans* has been legislated for the purpose of management and conservation as a single species^13,14^. Our results support a population structure consistent with the multi-species designation of Jackson and Groves^20^, with a Northern, Central and Southern species of greater glider. We are not able to resolve subspecies structure from our data.

While the three OTUs roughly align with the species designations described by Jackson^19^, there are some notable differences. In particular, the Central OTU that corresponds with the proposed “central” species, *P. armillatus*, extends much farther south than anticipated based on the distribution described in Jackson^19^. Although we had a relatively small, non-continuous sample, it is also worth noting that we found no evidence of the Southern OTU that corresponds with *P. volans* in Queensland, despite the range proposed by Jackson^19^ extending to Bundaberg. There is no information provided by Jackson^19^ to explain how the distributions in that text were determined. Notably, Jackson’s distribution for *P. volans* did not agree with the range proposed by his source, Arbogast^23^, who does not extend the distribution of *P. volans* into Queensland, and that agrees with our data.

An important caveat is that the findings from this study arose in the process of conducting unrelated research to look at relationships between variations in physiology and climate at the northern and southern distribution of what was thought to be one species at the time (McGregor et al., in prep). Given the distinct morphological differences between the northern and southern samples, we decided to use genetics to investigate the population structure. We did not comprehensively sample greater gliders throughout their range and no samples were obtained in New South Wales. Hypothesised species can represent arbitrary slices across geographic clines^33^, and we have been cautious in interpreting our data in the context of existing work that defines the three species and draws support from the SNP data^19,20^. In particular, we have not attempted to describe the new taxa de novo using genetic data alone, but rather to examine the genetic data to see if it is consistent or inconsistent with the taxonomic concepts put forward by Jackson and Groves^19,20^ of three species. Our data are certainly inconsistent with the samples representing panmictic or close to panmictic populations, and in the view of the magnitude of the observed fixed differences and the very substantial counts of private alleles held in each of our three OTUs, the genetic divergences we observed are very unlikely to be explained by punctuated sampling along a cline. Additional surveys should be undertaken to obtain a more complete understanding of genetic structure across the range of greater gliders. While our data does not enable an exhaustive assessment of *Petauroides* systematics, it does provide the first genetic evidence to support multiple extant species in *Petauroides*, and this finding has important conservation implications.

Over the past decade, several studies have reported alarming declines in greater glider populations in the Blue Mountains, NSW and Central Highlands, Vic and localized extinctions in other areas, such as Booderee NP, NSW^9–12^. Population declines have been attributed to the cumulative effects of land clearing and climate change, including logging, lower than average rainfall and increased occurrence of intense fires^11,34–36^. This year, the most destructive wildfire season on record burned through substantial portions of greater glider habitat in south-eastern Australia. The extent of greater glider habitat impacted by fire and repercussions to populations are still under investigation. However, as a result of the recent widespread habitat destruction and the history of unresolved population declines, the conservation status of the currently recognised single species, *P. volans*, is being reassessed.

The division of the greater glider into multiple species reduces the presumed, widespread distribution of *P. volans*, further increasing conservation concern for this species and highlighting the lack of information about the other *Petauroides* spp. Ecological studies have focused primarily on populations in NSW and Vic with only a few published studies of *P. minor*^16^ and no prior studies of *P. armillatus*. The knowledge that there is now genetic support for multiple species, with distributions that are probably much smaller than the previously designated range of *P. volans*, should be a consideration in future conservation status decisions and management legislation.

## Methods

### Sample collection

**Figure 4:**
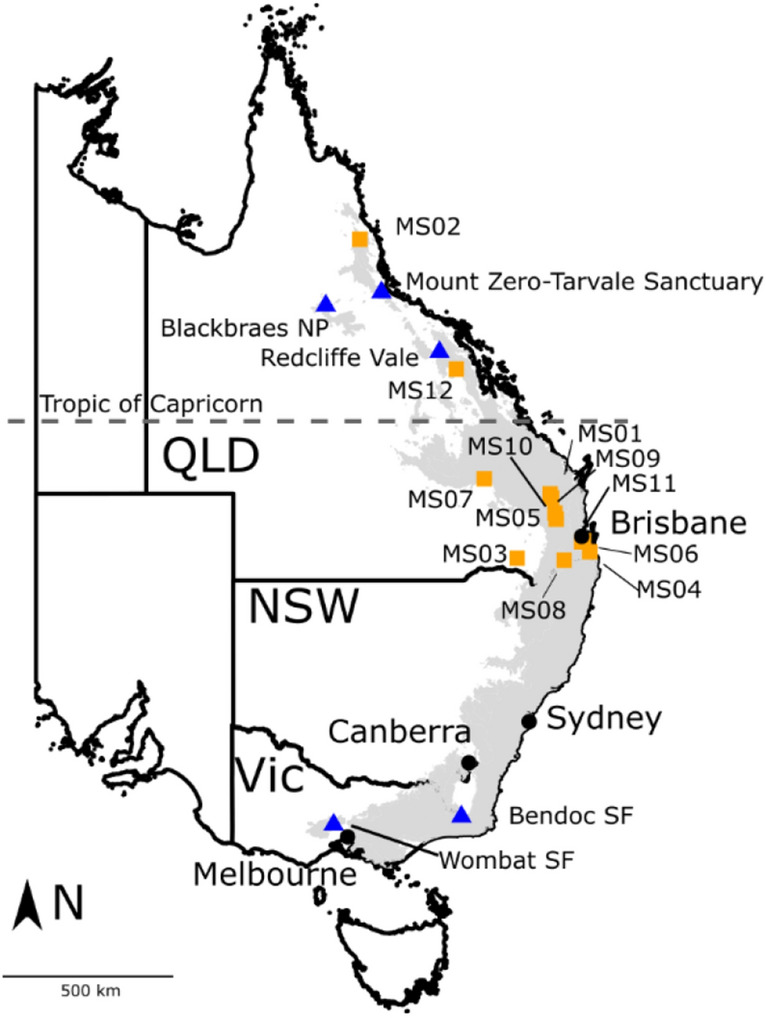
Location of the five study areas in eastern Australia in blue triangles (Blackbraes National Park (NP), Mount Zero-Taravale Sanctuary, Redcliffe Vale, Bendock State Forest (SF) and Wombat SF), and location of museum samples in orange squares (MS). The grey shading represents the current distribution of the greater glider from the Australian Species of National Environmental Significance Database^49^. This map was generated in R using the Australian coastline data from Geoscience Australia^50^ and multiple R packages (ggplot2^39^, ggsn^51^, sp^52,53^, rgdal^54^, raster^55^).

We sampled tissue from wild greater gliders from four locations that broadly represent the northern and southern distribution of the *Petauroides* latitudinal and longitudinal geographic range as part of a separate study to investigate relationships between animal physiology and climate (Fig. 4). The sites included Mount Zero-Taravale Australian Wildlife Sanctuary (19° 07′ 18″ S, 146° 04′ 42″ E, n = 18) and Blackbraes National Park (19° 34′ 39″ S, 144° 05′ 05″ E, n = 15) in North Queensland, and Bendoc State Forest (37° 10′ 35″ S, 148° 56′ 52″ E, n = 9) and Wombat State Forest (37° 29′ 50″ S, 144° 09′ 23″ E, n = 6) in Victoria. We then conducted additional field sampling in Redcliffe Vale, Queensland (21° 06′ 57″ S, 148° 06′ 58″ E, n = 18) (Fig. 4) as this was the suggested location of the proposed *P. armillatus*^19,20^. In addition, 12 museum specimen tissue samples were obtained from the Queensland Museum from greater gliders collected from northern, central and southern Queensland to investigate genetic structure in that area more broadly (Fig. 4). Additional information about the climate, geography and vegetation at each sample location can be found in Supplementary Material, (Table S1 online).

Wild greater gliders were located through spotlight searches using high-powered, handheld torches (Ledlenser P7, Zweibrüder Optoelectronics GmbH and Co., Solingen, Germany) to detect greater glider eye shine. Individuals were then captured using a gas-powered, tranquilizer dart-gun (Montech Black Wolf; Tranquil Arms Company, VIC, Australia) and darts specifically designed for mammals between 400 and 2000 g (0.5 ml; Minidarts, Tranquil Arms Company) containing 30–60 mg Zoletil 100 (Zolazepan and Tiletamine 50:50; Virbac), depending on the estimated body mass of the animal. While still under sedation, captured individuals were weighed and sexed, and reproductive status was assessed. External measurements were taken with vernier callipers using external jaws to measure head length (tip of snout to occipital bone protuberance), head width (widest part of one zygomatic arch to the analogous location on the other side of the head), ear width (widest part of the ear when flattened), ear length (from tragus to the outermost edge of the ear, excluding fur), and knee to heal-hind limb (top of knee to base of the heel with limb flexed to ninety degrees). Body length (occipital bone protuberance to the base of the tail, following the spine, with head in-line with the plane of the body) and tail length (cloaca to the tip of the tail, excluding fur) were measured with a flexible tape measure. Each individual was marked with a PIT tag (AVID Microchip Company, CA, USA) implanted subcutaneously and a tissue sample was clipped from the margin of the ear for DNA analysis. All work involving live animals complied with animal ethics and relevant guidelines and regulations. The animal capture and tissue collection was approved by James Cook University (Animal Ethics Permits A2137, A2545).

### Morphology data investigation

Principal components analysis of the eight measured morphological traits for greater gliders from the five sites (Taravale, Blackbraes, Redcliffe Vale, Bendoc, and Wombat) was performed in R, using the “prcomp” function. The plot was generated using the ggplot2 package^37^. We then used a canonical variate analysis (CVA) in R package MASS^38^ to analyse regional group structure (Northern, Central, and Southern) in the multivariate data. To explore whether there were significant differences in measured traits between sexes and account for unequal sample sizes, we used linear models with backward elimination variable selection method and Tukey’s post-hoc multiple comparison test. Linear models included each morphological trait as the response variable and region (Northern, Central, Southern), sex (male, female) and the interaction between region and sex. Insignificant variables were eliminated until only significant variables remained. Based on these results, sex was pooled and Tukey’s post-hoc multiple comparison test was used to compare measured morphological traits between regions. We also explored differences between sites with Tukey’s post-hoc multiple comparison test.

### DNA extraction and sequencing

DNA was extracted by Diversity Arrays Technologies (DArT Pty Ltd, Canberra, Australia) using a NucleoMag 96 Tissue Kit (MachereyNagel, Duren, Germany) coupled with NucleoMag SEP (Ref. 744900) to allow automated separation of high-quality DNA on a Freedom Evo robotic liquid handler (TECAN, Miinnedorf, Switzerland). Tissue was first incubated overnight with proteinase K, adjusted in concentration depending on the tissue. Sequencing for SNP genotyping was done using DArTseq (DArT Pty Ltd, Canberra, Australia), which uses a combination of complexity reduction using restriction enzymes, implicit fragment size selection and next generation sequencing^39^, as described in detail by Kilian et al.^40^ and Georges et al.^26^. Essentially, DArTseq is an implementation of sequencing complexity-reduced representations^41^ and more recent applications on next generation sequencing platforms^42,43^. To achieve the most appropriate complexity reduction (the fraction of the genome represented, controlling average read depth and number of polymorphic loci), four combinations of restriction enzymes (Pstl enzyme combined with either Hpall, Sphl, Nspl or Msel) were evaluated and restriction enzyme combination of Pstl (recognition sequence 5′-CTGCAIG-3′) and Sphl (5′-GCATGIC-3′) was selected.

Amplification using polymerase chain reaction (PCR)^26,44^ and the conditions applied are as described in Georges et al.^26^. After PCR, equimolar amounts of amplification products from each sample were pooled and applied to cBot (Illumina) bridge PCR for sequencing on the Illumina Hiseq 2500. The sequencing (single end) was run for 77 cycles to yield sequence tags of 20–69 bp after removing adaptors.

### SNP genotyping

Sequences generated from each lane were processed using proprietary DArT Pty Ltd analytical pipelines as described by Georges et al.^26^. In particular, one third of samples were processed twice from DNA, using independent adaptors, to allelic calls as technical replicates, and scoring consistency (repeatability) was used as the main selection criterion for high quality/low error rate markers. The DArT analysis pipelines have been tested against hundreds of controlled crosses to verify Mendelian behaviour of the resultant SNPs as part of their commercial operations. The resultant data set contained the SNP genotypes and various associated metadata of which CloneiD (unique identity of the sequence tag for a locus), repAvg (proportion of technical replicate assay pairs for which the marker score is identical), CallRate (proportion of individuals scored at a particular locus) and SnpPosition (position in the sequence tag at which the defined SNP variant base occurs) are of particular relevance to our analyses.

### Additional SNP filtering

The SNP data and associated metadata were read into a genlight object ({adegenet}^45^) to facilitate processing with package dartR (version 1.8)^46^. We first removed all but one SNP from each sequence tag (12,782 SNPs removed) and retained only those loci supported by a read depth between 5 × and 100 × (8895 loci removed). Three individuals were removed from the dataset owing to an exceptionally poor call rate of less than 0.5 (MS9, MS12, MS8) and resultant monomorphic loci removed from the dataset. Loci with a repeatability less than 0.99 were removed (2380 loci) and finally, loci with a call rate of less than 0.95 were removed (9870 loci). We regard the data remaining after this additional filtering (11,317 SNP markers for 75 individuals) as highly reliable.

### Visualization

Genetic similarity among individuals, populations and colour morphs was visualized using ordination (principal coordinates analysis or PCoA^47^) as implemented in the gl.pcoa and gl.pcoa.plot functions of dartR. A scree plot of eigenvalues guided the number of informative axes to examine^48^, taken in the context of the average percentage variation explained by the original variables (using the gl.pcoa.scree function in dartR).

### Diagnosable units

Diagnosable units are populations or aggregations of populations that can be diagnosed by one or more fixed allelic differences. Individuals that were intermediate between two aggregations in the PCoA (T1 and T5) were removed as suspected hybrids or introgressed individuals and examined separately. A fixed difference analysis as implemented in dartR was undertaken, and pairs of populations with no fixed allelic differences were progressively amalgamated to yield a putative set of diagnosable operational taxonomic units (OTUs). Putative OTUs that were distinctive because of false positives (owing to sampling error) were identified using gl.fixed.diff() with test = TRUE in dartR (see Georges et al.^26^, R scripts are provided in the online data repository), and pairs of populations for which the count of fixed differences did not exceed the estimated false positive rate were also amalgamated. Counts of private alleles were obtained from gl.report.pa in dartR.

### Genetic diversity

Expected heterozygosity was used as a measure of relative genetic diversity. Heterozygosity was obtained for each population from allele frequencies using the gl. report.heterozygosity function of dartR and pairwise comparisons of heterozygosity between populations were tested for significance using the gl.test.heterozygosity function in dartR (significance evaluated by re-randomizaton with 10,000 replicates).

### Hybridisation

The genotypes of suspected hybrids/introgressed individuals (T1 and T5) were examined using New Hybrids^28^ without specifying parental source populations. Briefly, New Hybrids uses simulation to characterize likelihood bins for each of the parental populations, F1 hybrids generated by crossing the parentals, F2 hybrids, and backcrosses between the F1 hybrids and the parental populations. These bins are used to estimate the likelihood of an individual belonging to each bin, and these likelihoods are rendered and scaled to produce posterior probabilities of bin membership. Parameters were set as ThetaPrior = Jeffreys, PiPrior = Jeffreys, burnin = 10,000, sweeps = 10,000 and the default genotype frequency classes (P0, P1, F1, F2, F1 × P0, F1 × P1). New Hybrids gives a posterior probability of individual membership in each of the genotype frequency classes, allowing effective assignment of first generation hybrids (F1), second generation hybrids (F2) and backcrosses of F1 hybrids to the parental populations.

### Relatedness

Two individuals with genotypes intermediate between Taravale/Blackbraes and Redcliffe Vale (T1 and T5) were found in the same cluster of trees in Taravale. Given their status and proximity, we wished to examine the possibility that they were in a parent–offspring relationship. We did this by assuming they were parent–offspring and examining the frequency of pedigree inconsistencies (e.g. one individual homozygous reference, the other homozygous alternate). Theoretically, the count of pedigree inconsistencies should be zero for true parent–offspring pairs, but errors in calling the SNPs generates false positives. To overcome this, we generated counts of pedigree inconsistencies for all pairs of individuals to generate a null expectation and identified putative pairs of parent–offspring as outliers using software to be included in the next release of dartR (gl.report.parent.offspring).

## Supplementary information

Supplementary Information.

## Data Availability

The datasets generated and analysed during the current study have been uploaded as related manuscript files that are available to the editors and reviewers. The raw data and all scripts we used for analysing it are publicly available through The Tropical Data Hub Research Data Repository (https://research.jcu.edu.au/researchdata) at https://doi.org/10.25903/xx67-2m46.
