# Author Correction: Genetic evidence supports three previously described species of greater glider, *Petauroides volans*, *P. minor*, and *P. armillatus*

**DOI:** 10.1038/s41598-021-99388-5

**Published:** 2021-10-04

**Authors:** Denise C. McGregor, Amanda Padovan, Arthur Georges, Andrew Krockenberger, Hwan-Jin Yoon, Kara N. Youngentob

**Affiliations:** 1 College of Science and Engineering, James Cook University, Cairns, QLD 4878 Australia; 2 CSIRO Black Mountain Science and Innovation Park, Canberra, ACT 2601 Australia; 3 Institute for Applied Ecology, University of Canberra, Canberra, ACT 2601 Australia; 4 Division of Research and Innovation, James Cook University, Cairns, QLD 4878 Australia; 5 Statistical Consulting Unit, Australian National University, Canberra, ACT 2601 Australia; 6 Research School of Biology, Australian National University, Robertson Building, 46 Sullivan’s Creek Road, Canberra, ACT 2601 Australia

Correction to: *Scientific Reports* 10.1038/s41598-020-76364-z, published online 06 November 2020

The original version of this Article and accompanying Supplementary Information file contained a repeated error, where the co-ordinates given for the Redcliffe Vale, Queensland study site “(21° 06′ 57″ S, 148° 06′ 58″ E, n = 18)” was incorrectly given as “(21° 06′ 57″ S, 148° 56′ 58″ E, n = 18).”

The original Article and accompanying Supplementary Information file have been corrected.

