# Supplementary material for: Genetic evidence supports three previously described species of greater glider, Petauroides volans, P. minor, and P. armillatus
Source: Sci Rep. 2020 Nov 6;10:19284. doi: 10.1038/s41598-020-76364-z (PMC7648813; doi:10.1038/s41598-020-76364-z)
Supplement: Supplementary file 1 — Supplementary Information. [file 41598_2020_76364_MOESM1_ESM.docx]

Supplementary Materials

**Genetic evidence supports three previously described species of Greater Glider, *Petauroides volans*, *P. minor*, and *P. armillatus***

Denise McGregor^1^, Amanda Padovan^2^, Arthur Georges^3^, Andrew Krockenberger^4^, Hwan-Jin Yoon^5^, Kara Youngentob^6^*

^1^ James Cook University, College of Science and Engineering, Cairns, QLD 4878

^2^ CSIRO Black Mountain Science and Innovation Park, Canberra, ACT 2601

^3^ University of Canberra, Institute for Applied Ecology, Canberra ACT 2601

^4^ James Cook University, Division of Research and Innovation, Cairns, QLD 4878

^5^ Australian National University, Statistical Consulting Unit, Canberra ACT 2601

^6^ Australian National University, Research School of Biology, Canberra ACT 2601

*Corresponding Author

Dr Kara Youngentob

Research School of Biology

Australian National University

Robertson Building, 46 Sullivan’s Creek Road

Canberra ACT 2601

Email: kara.youngentob@anu.edu.au

Phone: +61 477 761 298

Table S1. Additional site information from the five locations where wild greater gliders were captured to obtain tissue samples for DArT analyses.

| Study area | Taravale Sanctuary, QLD | Blackbraes National Park, QLD | Redcliffe Vale, QLD | Bendoc State Forest, Vic | Wombat State Forest, Vic |
| --- | --- | --- | --- | --- | --- |
| Central coordinates | 19°07’18”S, 146°04’42”E | 19°34′39”S, 144°05′05”E | 21° 06’ 57”S, 148° 06’ 58”E | 37°10’35”S, 148°56’52”E | 37°29’50”S, 144°09’23”E |
| Elevation | 618-724 m above sea level | 1040-1065 m above sea level | 372-415 m above sea level | 810-930 m above sea level | 618-639 m above sea level |
| Bioregion | Einasleigh Uplands | Gulf Pains | Brigalow Belt North | Highlands Far East | Central Victorian Uplands |
| Temperatures  (Australian Bureau of Meteorology) | Average minimum 6.7°C (July) and average maximum 33.6°C (December) | Average minimum 5.3°C (July) and average maximum 37.2°C (November) | Average minimum 9.1°C (July) and average maximum 34.1°C (December) | Average minimum 4.9°C (July) and average maximum 25.5°C (January) | Average minimum 2.4°C (July) and average maximum 26.9°C (January) |
| Rainfall  (Australian Bureau of Meteorology) | Averages 1099 mm annually, with 80% occurring in the wet season (November-April) | averages 789 mm annually, with 86% occurring in the wet season (November-March) | Averages 697 mm annually, with 75% occurring in the wet season (November-March) | Averages 1187 mm annually, with 55% occurring in winter and spring (June-November) | Averages 936 mm annually, with 62% occurring in winter and spring (June-November) |
| Geology^1^ | Diverse terrain but fieldwork primarily located on alluvial soil valleys between rugged igneous ranges with granite outcrops. | Mesozoic sandstone plateaus, with red, yellow, and brown sandy soils. Undulating igneous hills with basalt outcrops. | Undulating to rugged ranges of igneous rock with alluvial plains. | Upland areas with Ordovician sedimentary bedrock and moderate to steep slopes and scarps comprised of Devonian granitic massifs and large granitic tors. The soil is primarily brown and red porous earths (Dermosols). | Straddles the great dividing range with steeply sloped hills and ridges. The geology is primarily Ordovician sediments and Quaternary basalts of volcanic origin. |
| Vegetation | Open eucalypt forests and woodlands dominated by multiple and varied canopy species including *Eucalyptus creba, E. tereticornis, E. portuensis, and Corymbia spp.* Common understory vegetation includes *Allocasurina spp*., *Acacia spp*. and *Grevillea spp.* | Open eucalypt forests and woodlands dominated by *Eucalyptus crebra*, *Corymbia citridora,* and less commonly, *E. acemenoides*. *Grevillea spp.* and *Acacia spp.* are common in the understory. | Open eucalypt forests and woodlands dominated by *Eucalyptus crebra* and *Corymbia citridora.* Common understory species include *Petalostigma pubescens* and *Acacia spp*. | Montane eucalypt forests dominated by *Eucalyplus dalrypleana*, *E. dives*, *E. panora*, *E. rubida*, and occasionally *E. obliqua*. Common understory species include *Daviesia ulicifolia*, *Leptospermum grandifolium*, and *Tasmannia lanceolata* | Tall-open eucalypt forests dominated by *Eucalyptus obliqua* and *E. radiata,* with *E. viminalis* and *E. ovata* also common. The understory is dominated by *Acacia dealbata* and *A. melanoxylon.* |

^1^David Johnson & Robert Henderson. *The Geology of Australia* (Cambridge University Press, 2016).

Table S2. Mean body size and fur measurements from captured wild greater gliders from the southern, central and northern regions. Only morphological data for adults are presented in this table. Mass unit is in kilograms, body length and tail length are measured in centimetres and all other measured traits are in millimetres.

| Location | Region | N | Sex | Mass | Head length | Head width | Body length | Tail length | Knee-to-heel length | Ear length | Ear width | Pelage colouration |
| --- | --- | --- | --- | --- | --- | --- | --- | --- | --- | --- | --- | --- |
| Bendoc | Southern | 6 | F | 1.42 | 67.30 | 39.42 | 37.42 | 51.75 | 121.70 | 41.60 | 32.44 | 5 Black with white underside.  1 Grey and white with white underside. |
| Bendoc | Southern | 2 | M | 1.37 | 63.85 | 38.50 | 37.75 | 48.75 | 120.90 | 39.98 | 31.75 | Black with white underside. |
| Wombat | Southern | 5 | F | 1.31 | 67.31 | 38.98 | 35.10 | 53.30 | 121.39 | 42.22 | 28.60 | 4 Black with white underside.  1 Grey and white with white underside. |
| Wombat | Southern | 1 | M | 1.24 | 70.60 | 39.50 | 30.00 | 50.00 | 115.60 | 42.05 | 32.90 | Black with white underside. |
| Redcliffe | Central | 8 | F | 0.88 | 63.76 | 36.98 | 28.75 | 45.34 | 114.05 | 41.02 | 30.14 | Brownish-silver back. Dark brown face, legs and tail. Cream underside. |
| Redcliffe | Central | 7 | M | 0.85 | 64.63 | 37.59 | 27.71 | 44.64 | 110.93 | 39.63 | 29.59 | Brownish-silver back. Dark brown face, legs and tail. Cream underside. |
| Blackbraes | Northern | 5 | F | 0.65 | 57.69 | 33.83 | 26.20 | 37.70 | 98.51 | 34.59 | 27.00 | Brownish-grey with cream underside. |
| Blackbraes | Northern | 5 | M | 0.64 | 53.85 | 33.56 | 26.80 | 35.10 | 101.87 | 34.51 | 24.32 | Brownish-grey with cream underside. |
| Taravale | Northern | 12 | F | 0.63 | 58.04 | 34.16 | 29.21 | 41.33 | 102.91 | 34.44 | 26.84 | Brownish-grey with cream underside. |
| Taravale | Northern | 5 | M | 0.68 | 58.11 | 35.23 | 28.40 | 40.60 | 102.50 | 35.41 | 22.32 | Brownish-grey with cream underside. |

Table S3. Museum specimens

| ID for DArT | Registration Number | Specimen Category Notes Count | Locality name | Field Coll Latitude | Field Coll Longitude | Field Coll Date | Field Coll Specimen Category |
| --- | --- | --- | --- | --- | --- | --- | --- |
| MS01 | A003170 | JM15443 | Murgon, inland and slightly above Sunshine Coast | -26.14 | 151.56 | 6-Apr-98 | DNA tissue |
| MS02 | A010356 /A010537 | JM20019, muscle | Atherton, N Qld | -17.22 | 145.37 | 25-Nov-12 | DNA tissue |
| MS03 | A003290 / A003291 | JM15576 | Killarney, 831 Condamine River Road, inland below Gold Cast, east Warwick | -28.13 | 150.475 | 30-Mar-03 | DNA tissue |
| MS04 | A007279 | JM19593, liver | Currumbin area, SEQ, coastal between Gold Coast and Coolangatta | -28.08 | 153.29 | 21-Aug-92 | DNA tissue |
| MS05 | A014891 / A014901 | JM21082 | Benarkin, Williams Road, inland south of Sunshine Coast | -26.523 | 152.082 | 13-Mar-16 | DNA tissue |
| MS06 | A003167/A003168 | JM15441 | Forestdale, Johnson Road, below Brisbane | -27.39 | 153 | 31-May-96 | DNA tissue |
| MS07 | A014962 /A014963 | JM21112, liver | Lake Murphy Regional Park, inland from Hervey Bay | -25.485 | 149.66 | 17-Apr-14 | DNA tissue |
| MS08 | A013139 / A013140 | JM20426, skin | Spring Creek Mountain, Carrs Lookout, inland below Gold Coast | -28.293 | 152.417 | 15-Jun-07 | DNA tissue |
| MS09 | A006017 /A006018 | JM15102 | Boat Mountain via Murgon, inland from Sunshine Coast | -26.09 | 150.4747 | 6-Jan-01 | DNA tissue |
| MS10 | A003169 | JM15442 | Nanango State Forest, inland from Sunshine Coast | -26.4 | 152.06 | Aug-97 | DNA tissue |
| MS11 | A009479 | JM15440 | Compton Road, Karawatha, below Brisbane | -27.37 | 153.05 | 8-May-95 | DNA tissue |
| MS12 | A010268 | JM19992 | Nebo, southeast of Mackay | -21.427 | 148.415 | 25-Jan-12 | DNA tissue |


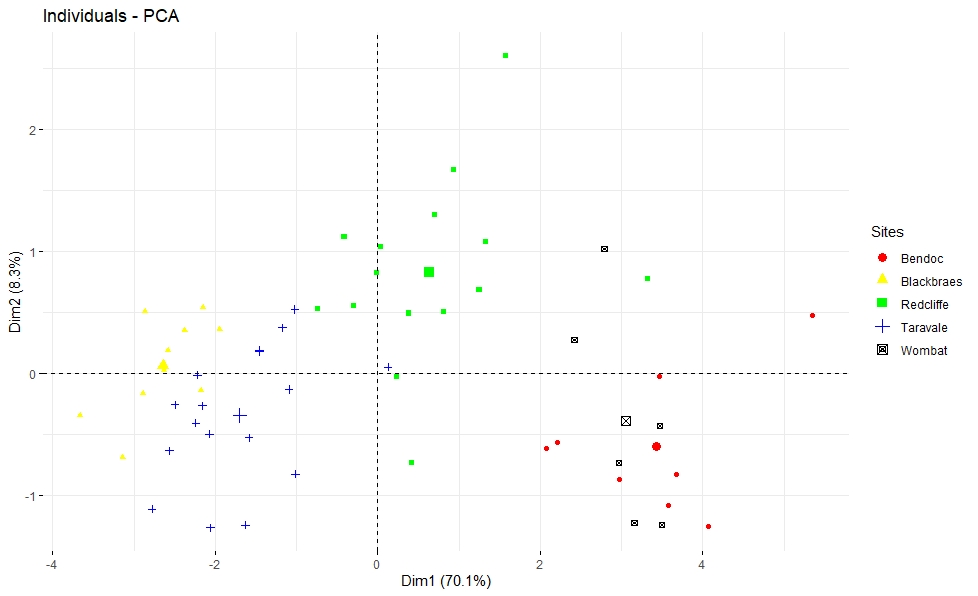

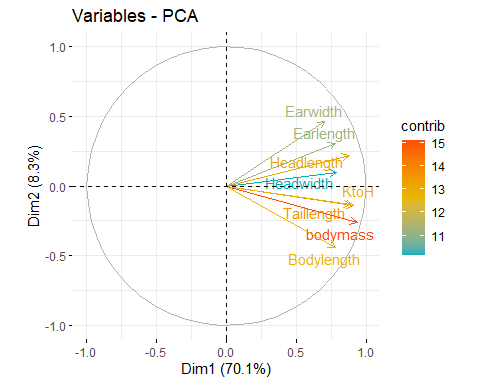


B

A

Figure S1. A) Principal component analysis (PCA) of the eight measured morphological traits coloured by sites (Northern sites are Taravale and Blackbraes, Central site is Redcliffe Vale, Southern sites are Wombat and Bendoc). PC1 (Dim1) explains 70.1% of the variation in the dataset and PC2 (Dim2) explains 8.3% of the variation. B) The contributions of the measured attributes.

Table S4 Latent vectors (loadings) for the principle component analysis of body measurements of 56 greater gliders from 5 sites

| Trait | PC1 | PC2 | PC3 | PC4 | PC5 | PC6 | PC7 | PC8 |
| --- | --- | --- | --- | --- | --- | --- | --- | --- |
| Body mass | 0.3959 | -0.3222 | 0.7415 | -0.0453 | -0.5439 | -0.1221 | -0.1085 | 0.8380 |
| Head length | 0.3711 | 0.2669 | -0.1802 | -0.0803 | 0.4039 | 0.7616 | 0.0469 | 0.0822 |
| Head width | 0.3342 | 0.1232 | -0.4425 | -0.6447 | -0.4450 | -0.1382 | 0.1624 | -0.1343 |
| Body length | 0.3290 | -0.5388 | 0.4958 | -0.2710 | -0.3074 | 0.3379 | 0.1200 | -0.3631 |
| Tail length | 0.3841 | -0.1704 | -0.2117 | 0.0768 | 0.2349 | -0.2116 | -0.7480 | -0.3366 |
| Knee to heel | 0.3750 | -0.1667 | -0.1481 | 0.2331 | 0.4410 | -0.3890 | 0.6186 | -0.1636 |
| Ear length | 0.3296 | 0.3747 | 0.-1165 | 0.6794 | -0.5242 | 0.0287 | 0.0108 | 0.0069 |
| Ear width | 0.2979 | 0.5676 | 0.6633 | -0.2307 | 0.1300 | -0.2736 | -.0551 | -0.0322 |
| Standard deviation | 2.3674 | 0.8132 | 0.7495 | 0.6538 | 0.5821 | 0.4235 | 0.4066 | 0.2480 |
| Proportion of variance | 0.7006 | 0.0827 | 0.0702 | 0.0534 | 0.0424 | 0.0224 | 0.0207 | 0.0078 |
| Cumulative proportion | 0.7007 | 0.7832 | 0.8535 | 0.9069 | 0.9492 | 0.9717 | 0.9923 | 1.000 |

Table S5. Tukey’s Post-hoc test between the two Northern locations (Taravale and Blackbraes) and the two

Southern locations (Wombat and Bendoc) for each measured morphological trait. Mass unit is in kilograms,

body length and tail length are measured in centimetres and all other measured traits are in millimetres.

| Comparison | Taravale - Blackbraes | | | Wombat - Bendoc | | |
| --- | --- | --- | --- | --- | --- | --- |
| Trait | Diff. in mean (SE) | t value | P value | Diff. in mean (SE) | t value | P value |
| Body mass | 0.065 (0.0386) | 1.680 | 0.450 | -0.109 (0.0523) | -2.086 | 0.238 |
| Head length | 2.292 (1.127) | 2.034 | 0.261 | 1.421 (1.527) | 0.930 | 0.882 |
| Head width | 0.779 (0.783) | 0.994 | 0.854 | -0.121 (1.061) | -0.114 | 1.000 |
| Body length | 2.470 (1.045) | 2.365 | 0.139 | -3.250 (1.416) | -2.295 | 0.160 |
| Tail length | 4.718 (0.945) | 4.993 | < 0.0001 | 1.750 (1.281) | 1.367 | 0.646 |
| Knee to heel | 2.601 (1.871) | 1.390 | 0.632 | -1.075 (2.536) | -0.424 | 0.993 |
| Ear length | 0.177 (1.212) | 0.146 | 1.000 | 0.998 (1.643) | 0.607 | 0.973 |
| Ear width | -0.148 (1.223) | -0.121 | 1.000 | -2.952 (1.657) | -1.781 | 0.390 |


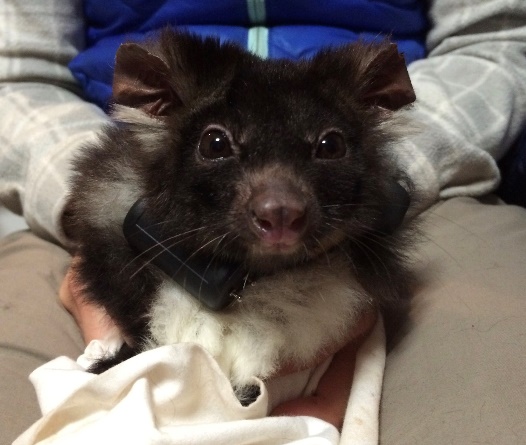

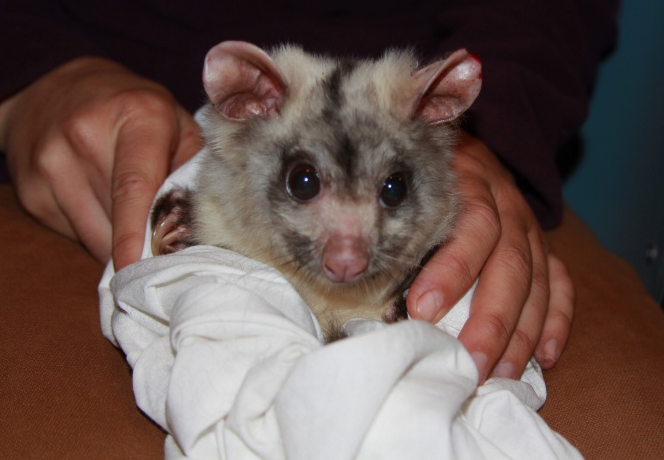

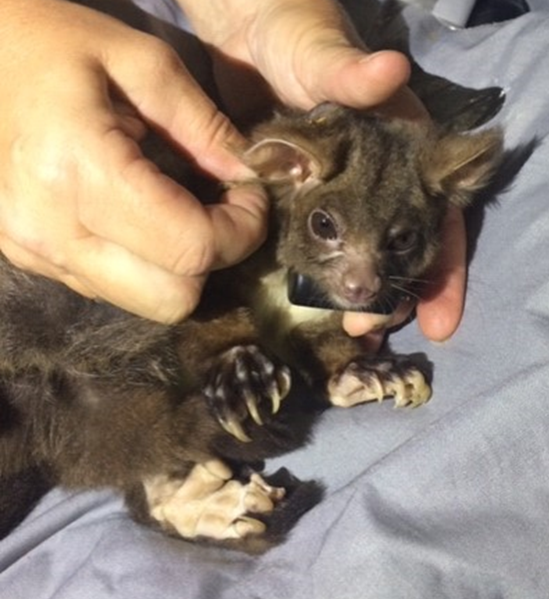

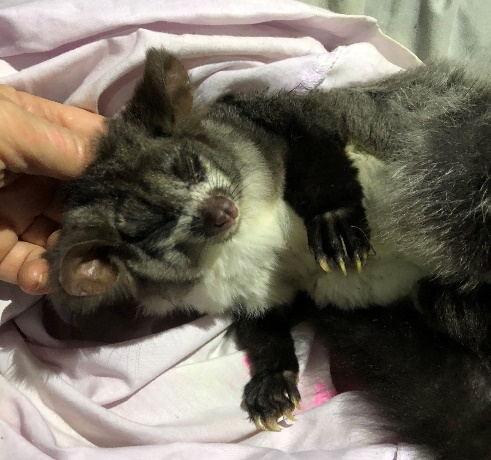


Figure S2. Images of captured wild greater gliders. The larger size of *Petauroides volans* (right, showing light and dark colour phases) compared

to the proposed northern *P. minor* (top left) and central *P. armillatus* (bottom left) is evident with animals in hand. Photos by Denise McGregor
